# Cryptochrome 1 as a state variable of the circadian clockwork of the suprachiasmatic nucleus: Evidence from translational switching

**DOI:** 10.1073/pnas.2203563119

**Published:** 2022-08-17

**Authors:** David McManus, Lenka Polidarova, Nicola J. Smyllie, Andrew P. Patton, Johanna E. Chesham, Elizabeth S. Maywood, Jason W. Chin, Michael H. Hastings

**Affiliations:** ^a^Neurobiology Division, Medical Research Council Laboratory of Molecular Biology, Cambridge CB2 0QH, United Kingdom;; ^b^PNAC Division, Medical Research Council Laboratory of Molecular Biology, Cambridge, CB2 0QH, United Kingdom

**Keywords:** genetic code expansion, noncanonical amino acid, feedback, oscillator, transcriptional inhibition

## Abstract

The suprachiasmatic nucleus (SCN) of the hypothalamus is the principal clock driving circadian rhythms of physiology and behavior that adapt mammals to environmental cycles. Disruption of SCN-dependent rhythms compromises health, and so understanding SCN time keeping will inform management of diseases associated with modern lifestyles. SCN time keeping is a self-sustaining transcriptional/translational delayed feedback loop (TTFL), whereby negative regulators inhibit their own transcription. Formally, the SCN clock is viewed as a limit-cycle oscillator, the simplest being a trajectory of successive phases that progresses through two-dimensional space defined by two state variables mapped along their respective axes. The TTFL motif is readily compatible with limit-cycle models, and in *Neurospora* and *Drosophila* the negative regulators Frequency (FRQ) and Period (Per) have been identified as state variables of their respective TTFLs. The identity of state variables of the SCN oscillator is, however, less clear. Experimental identification of state variables requires reversible and temporally specific control over their abundance. Translational switching (ts) provides this, the expression of a protein of interest relying on the provision of a noncanonical amino acid. We show that the negative regulator Cryptochrome 1 (CRY1) fulfills criteria defining a state variable: ts-CRY1 dose-dependently and reversibly suppresses the baseline, amplitude, and period of SCN rhythms, and its acute withdrawal releases the TTFL to oscillate from a defined phase. Its effect also depends on its temporal pattern of expression, although constitutive ts-CRY1 sustained (albeit less stable) oscillations. We conclude that CRY1 has properties of a state variable, but may operate among several state variables within a multidimensional limit cycle.

## Significance

Circadian clocks adapt us to our rhythmic world, setting the tempo to our lives. Their disruption (e.g., by shiftwork) therefore carries a severe cost in health. The suprachiasmatic nucleus (SCN) is the principal brain clock of mammals, its time keeping pivoting around a delayed negative feedback loop of gene and protein expression. By using “translational switching” as a means to reversibly control the expression of the negative feedback regulator Cryptochrome 1 (CRY1) in SCN organotypic slices, we show that acute changes in the level of CRY1 define circadian time. We thereby bridge theoretical and biochemical perspectives of the SCN clockwork.

Circadian clocks drive daily rhythms of physiology and behavior that adapt organisms to environmental cycles (1–3). The suprachiasmatic nucleus (SCN) of the hypothalamus is the principal circadian clock of mammals (4), coordinating innumerable cellular clocks distributed across the body (5). It is entrained by direct retinal input, such that SCN-defined circadian time is predictive of solar time. In constant conditions its autonomous time keeping persists indefinitely. The temporal organization imposed by the SCN is pivotal for mental and physical health (6, 7) and its disruption carries a heavy burden (8–10).

At a cell-autonomous level, circadian clocks are self-sustaining transcriptional/translational feedback loops (TTFLs), whereby negative regulatory complexes inhibit transcription of their cognate genes. In the fungus *Neurospora crassa*, the negative and positive factors are Frequency (FRQ) and the White Collar (WC) proteins, respectively (11). In *Drosophila melanogaster*, Clock and Cycle transactivate the *period* (*per*) and *timeless* (*tim*) genes, which encode the negative regulators Per and Tim (12). In mammals, CLOCK and BMAL1 are the positive regulators driving *Per1* and *Per2* and *Cryptochromes* (*Cry1* and *Cry2*) via E box regulatory sequences. PER and CRY proteins close the feedback loop by inhibiting those E boxes (13).

Circadian clocks can be viewed formally as attracting limit-cycle oscillators, which have the properties of stable, self-sustained oscillation, phase shifts in response to entraining cues, and a singularity point where oscillation stops (14–16). The simplest limit cycle is a trajectory that progresses through two-dimensional space, with successive phases defined by the instantaneous values of two state variables (interactive clock components) mapped along their respective orthogonal axes. Alternatively, the cell-autonomous clock may be a damped oscillator driven by noise, rather than a self-sustained noisy oscillator (17). Furthermore, it has been suggested that they operate close to a bifurcation that separates these states (18). Even if the stable limit-cycle model is correct, in practice, circadian clocks will likely consist of multidimensional limit cycles defined by several state variables. Nevertheless, the model offers a framework to understand circadian properties at the organismal level (19, 20), including how light and behavioral cues direct human free-running rhythms and experimental entrainment (21), and predict maladaptation to “real-life” rotational shiftworking (22).

The delayed negative feedback motif is directly compatible with a limit-cycle model, stimulating efforts to generate quantitative models of the TTFL (20). Indeed, real-time monitoring of the TTFL using genetically encoded reporters for circadian transcriptional activation and protein abundance has generated evidence for the presence of limit-cycle behavior in cultured mammalian cells (23) and informed the development of both deterministic and stochastic models of the mammalian TTFL (24, 25). Nevertheless, the state variables that define the mammalian clock remain poorly understood, in part because simply determining the effects of the presence or absence of a factor is not a sufficient test. Rather, a state variable is a “clock component whose rhythmic changes in abundance or activity, not mere presence in the cell, is a necessary element of the timekeeping mechanism” (26).

The identification of state variables therefore requires experimental control of their expression in a reversible and dose-dependent manner. This has been achieved in *Neurospora* using an inducible FRQ-encoding transgene (tg-*frq*) (27), constitutive expression of which directly inhibited circadian transcription of endogenous *frq*. Conversely, in arrhythmic *frq*-null strains, constitutive expression of tg-FRQ could not initiate circadian conidiation rhythms, but it did so when expressed in a circadian manner, consistent with a state-variable role. More significantly, not only did sustained overexpression of tg-FRQ suspend the FRQ-dependent rhythm but, on withdrawal of tg-FRQ, the conidiation rhythm resumed with a phase dictated by the time of withdrawal, namely the point at which the expression of endogenous *frq* is released from its nadir imposed by tg-FRQ. These results established “*frq* as encoding a central component, a state variable, in a cellular circadian oscillator” (27). A comparable approach using heat shock–inducible tg-Per (28) indicated that Per may be a state variable of the fly TTFL (29) (but see ref. 30). In mammals, modeling and experimental evidence suggest that rhythmic PER (but not CRY) is a nodal point for feedback that sustains rhythmicity (31, 32) and that CRY1 confers robustness but not oscillation (33). Equally, a series of knockdown and overexpression studies have shown various roles of TTFL components for normal function (34). Nevertheless, such studies are steady-state interventions, commonly in cell lines with labile circadian phenotypes. The aim of the current study was to test the potential role of CRY1 as a state variable of the SCN clockwork. To do this, we expressed tg-CRY1 under temporally specific promoters and also in a dose-dependent and reversible manner using translational switching (ts) (35) in which translation of tg-CRY1 is dependent on provision of a noncanonical amino acid.

## Results

### Constitutive CRY1 Expression Can Sustain Imprecise Circadian Rhythms in *Cry*-Null SCN.

**Fig. 1.**
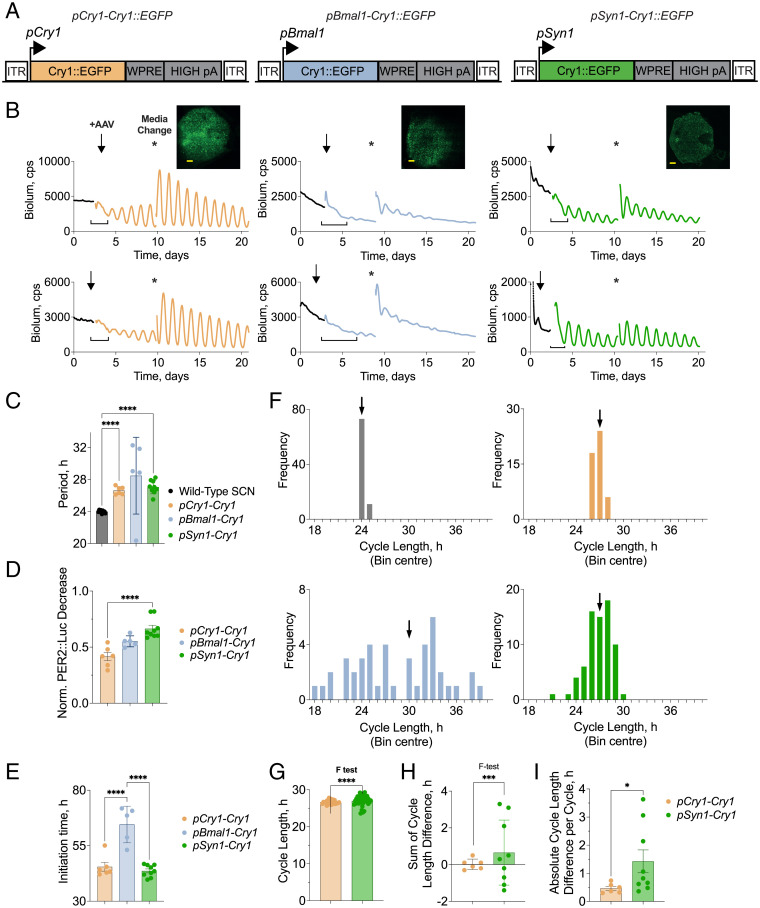
Constitutive CRY1 expression can sustain imprecise circadian rhythms in *Cry*-null SCN. (*A*) Schematic view of constructs used to express tg-CRY1::EGFP via AAV transduction, under three different minimal promoters: orange, *pCry1*; light blue, *pBmal1*; green, *pSyn1*. ITR, inverted terminal repeat; WPRE, Woodchuck Hepatitis Virus Posttranscriptional Regulatory Element. (*B*) Representative traces (two per construct) of PER2::Luc bioluminescence from *Cry1/Cry2*-null SCN slices before (black line) and after (colored line) transduction with AAVs encoding tg-CRY1::EGFP driven by different promoters (*Left*, *pCry1*, *n* = 6; *Center*, *pBmal1*, *n* = 5; *Right*, *pSyn1*, *n* = 9). After several days of recording, slices were transduced with AAV (black arrows). Seven to 8 d posttransduction, medium was changed (asterisks) and subsequent oscillation was recorded. Images show fixed slices with EGFP signal confirming successful transduction. cps, counts per second. (Scale bars, 100 µm.) (*C*) Group data (mean ± SEM) showing period of wild-type SCN (*Left*) or *Cry1/Cry2*-null SCN following transduction with AAVs encoding tg-CRY1::EGFP. Period of the oscillation was measured following medium change. (*D* and *E*) Group data (mean ± SEM) showing acute decrease of baseline PER2::Luc bioluminescence (*D*) and time to initiate circadian rhythms (*E*) following transduction of *Cry1/Cry2*-null SCN with AAVs encoding tg-CRY1::EGFP. Both measurements take the start point of initiation as the last bioluminescence recording pretransduction and the end point as the trough of the first restored oscillation (square brackets in *B*). (*F*) Histograms showing frequency distribution of cycle-to-cycle period of wild-type SCN or *Cry1/Cry2*-null SCN following transduction with AAVs encoding tg-CRY1::EGFP. Bin widths of 1 h. Data show the number of cycles from all slices within each cohort grouped into 1-h bins. A larger number of cycle lengths across the cohort in fewer bins shows a more stable rhythm. Black, wild-type SCN; orange, light blue, and green: *Cry*-null SCN with *pCry1-Cry1::EGFP*, *pBmal1-Cry1::EGFP*, and *pSyn1-Cry1::EGFP*, respectively. Arrows show the bin containing the group mean period shown in *C*. (*G*) SD of cycle-to-cycle lengths across the whole cohort of SCN showing variability in circadian period across all slices of *Cry1/Cry2*-null SCN following transduction with AAVs encoding *pCry1*- or *pSyn1*-driven tg-CRY1::EGFP. Cycle lengths across the entire cohort were much less stable for SCN transduced with *pSyn1*-driven CRY1::EGFP than *pCry1*-driven CRY1::EGFP, as demonstrated by a significant difference in group variance shown by an *F* test. F value (F), and degrees of freedom (DFn, Dfd): 6.870, 35, 23; *P* < 0.0001. (*H*) Sum of consecutive cycle length differences for six cycles postmedia change. The mean sum of consecutive cycle length differences of rhythms restored by *pCry1*-driven tg-CRY1::EGFP expression is not significantly different from those restored by *pSyn1*-driven tg-CRY1::EGFP (unpaired *t* test, *P* = 0.4), and neither are significantly different from zero (one-sample *t* test: *pCry1*, *P* = 0.89; *pSyn1*, *P* = 0.30). However, the variance between the two groups is significant (*F* test: *pCry1* vs. *pSyn1*, F, DFn, Dfd = 38.39, 8, 5; *P* = 0.0009, as shown). (*I*) Group data showing the absolute cycle length difference per cycle across six cycles of rhythms restored by *pCry1*- and *pSyn1*-driven tg-CRY1::EGFP expression. The average period correction per cycle is significantly larger in the SCN slices transduced with AAV1-*pSyn1-Cry1* than AAV1-*pCry1-Cry1 (P* = 0.046, Welch’s *t* test). The variance is also significantly larger in the AAV1-*pSyn1-Cry1* slices (F, DFn, Dfd = 49.94, 8, 5; *P* = 0.0005). *P < 0.05, **P < 0.01, ***P < 0.001, ****P < 0.0001.

The temporal pattern of expression of a state variable determines its efficacy within a limit cycle. We tested this for tg-CRY1 by examining the initiation of circadian rhythmicity in *Cry1/Cry2*-null SCN slices when tg-CRY1, with an enhanced green fluorescent protein (EGFP) tag, was expressed from adeno-associated viral vectors (AAVs) under different temporally specific promoters (Fig. 1*A*). Post hoc, confocal microscopy of fixed slices confirmed expression of tg-CRY1 via the EGFP tag. Bioluminescence from the PER2::Luciferase (PER2::Luc) circadian reporter was recorded for several days to confirm arrhythmicity before transduction with AAVs (Fig. 1*B*). After transduction with AAV in which tg-CRY1::EGFP was driven by the minimal circadian *Cry1* promoter (AAV1-*pCry1*-*Cry1::EGFP*) which peaks at the beginning of circadian night (36), high-amplitude circadian oscillations were initiated within ∼48 h and were sustained after AAV washout by medium change (Fig. 1 *B*, *Left*). In contrast, tg-CRY1 driven by the antiphasic *pBmal1* promoter which peaks in late circadian night (37) (AAV1-*pBmal1*-*Cry1::EGFP*) initiated extremely weak rhythms (Fig. 1 *B*, *Center*). Transduction of SCN with AAV in which tg-CRY1 was driven by the constitutively active (see below) *pSyn1* promoter (AAV1-*Syn1-Cry1::EGFP*) also caused rhythmic bioluminescence rhythms, albeit less well defined (Fig. 1 *B*, *Right*). Following change of medium 7 d after transduction, the period sustained by rhythmic *pCry1*-driven tg-CRY1 (26.6 ± 0.2 h) was not significantly different from that seen with *pSyn1*-driven tg-CRY1 (26.9 ± 0.3 h), and both were significantly longer than the ∼24-h period of wild-type SCN (24.1 ± 0.1 h) (Fig. 1*C*). This long period is characteristic of a *pCry1*-driven circadian rhythm in a *Cry2*-null SCN [26.2 ± 0.1 h in the genomic *Cry2-null* mutant (38)]. The very weak rhythms driven by *pBmal-Cry1* had a longer period oscillation but were more variable between slices (29.9 ± 0.8 h) (Fig. 1*C*). We next compared the behavior of the oscillations and assessed the promoter-dependent quality of restored rhythms. The acute decline (square brackets, Fig. 1*B*) in bioluminescence following transduction, consistent with the initiation of negative feedback by tg-CRY1 on PER2 expression, was greater with constitutive tg-CRY1 expression than with the early or late rhythmic promoters (initial normalized PER2::Luc decrease: *pCry1-Cry1*: 0.42 ± 0.04; *pBmal1-Cry1*: 0.55 ± 0.02; *pSyn1-Cry1*: 0.66 ± 0.3; *P* = 0.0002, one-way ANOVA) (Fig. 1*D*). There was also a marked difference in the time taken for tg-CRY1 to initiate stable rhythms, with *pCry1-Cry1* (initiation time, 45.5 ± 2.0 h) and *pSyn1-Cry1* (43.7 ± 0.9 h) being more rapidly effective than when tg-CRY1 was driven by *pBmal1* (64.6 ± 3.7 h) (*P* < 0.0001, one-way ANOVA) (Fig. 1*E*).

A hallmark of circadian rhythms is the robustness of oscillation with little cycle-to-cycle variation about the mean period. Indeed, when plotted as a frequency distribution, all individual cycle lengths from the cohort of wild-type SCN showed little variation about their mean period (Fig. 1 *F*, *Top Left*, arrow shows bin containing mean period plotted in Fig. 1*C*). In contrast, much greater variation is seen across all *Cry1/Cry2*-null SCN transduced with AAV1-*pBmal1-Cry* (Fig. 1 *F*, *Top Right*) with no obvious consensus period. Therefore, tg-CRY1 expressed rhythmically but at the wrong time in the cycle is unable to recapitulate the precise oscillations of a wild-type SCN. The key comparison is between *pCry1*- and *pSyn1*-driven tg-CRY1. Oscillations were restored to *Cry1/Cry2*-null SCN transduced with either AAV but, importantly, only *pCry1-*driven tg-CRY1 could sustain precise circadian rhythms. The variation in cycle lengths across all SCN transduced with AAV1 *pCry1-Cry1* (Fig. 1 *F*, *Bottom Left*) shows similar distributions to the cycle lengths in wild-type slices, whereas greater variation is seen in rhythms generated following transduction with AAV1-*pSyn1-Cry1*. Moreover, although both *pSyn1-* and *pCry1-Cry1* restore oscillations of the same mean period, there is significantly greater variation in the cycle length of *pSyn1*-driven tg-CRY1 when compared with those from *pCry1* (Fig. 1*G*; *F* test, *P* < 0.0001). To further assess the quality of the oscillations, we examined the sum of consecutive cycle length differences for SCN transduced with both AAV1-*pCry1-Cry1* and AAV1-*pSyn1-Cry* (39). This revealed a significantly larger variation in the *pSyn1-*driven tg-CRY1 SCN (Fig. 1*H*; *F* test, *P* = 0.0005). Equally, *pSyn1*-driven tg-CRY1 SCN showed a significantly larger cycle-to-cycle change than did *pCry1-*driven (Fig. 1*I*; Welch’s *t* test, *P* = 0.046). Constitutive expression of *pSyn1* tg-CRY1 can therefore sustain rhythmicity, but does so with less precision and greater variance in a cycle-to-cycle period. Thus, constitutive expression of tg-CRY1 could sustain SCN rhythms but not as effectively as did correctly phased circadian expression of tg-CRY1. To pursue further the putative role of CRY1 as a state variable, we next tested the effect of tg-CRY1 expression on a competent SCN oscillator.

### Constitutive, but Not Rhythmic, Expression of CRY1 Compromises the Circadian Oscillator of Wild-Type SCN.

**Fig. 2.**
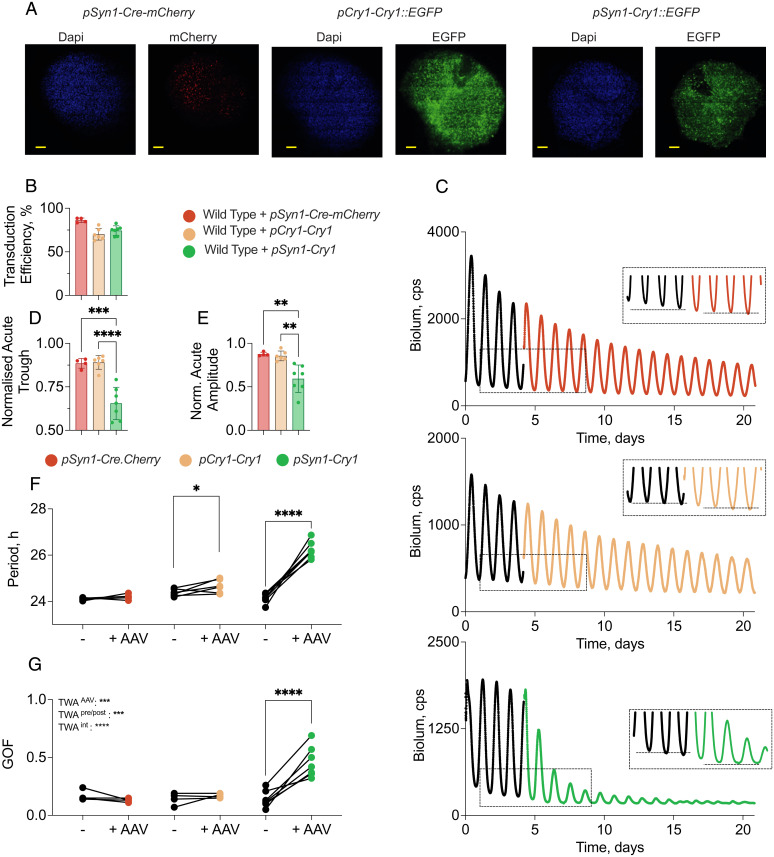
Constitutive, but not rhythmic, expression of CRY1 compromises the circadian oscillator of wild-type SCN. (*A*) Representative confocal images of wild-type SCN slices transduced with AAV expressing Cre::mCherry under *pSyn1* (*Left*), tg-CRY1::EGFP under the minimal *pCry1* promoter (*Center*), or the constitutive neuronal *pSyn1* promoter (*Right*). (Scale bars, 100 µm.) (*B*) Transduction efficiency (mean and individual SCN values) of *pCry1-Cry1::EGFP*, *pSyn1*-*Cry1::EGFP*, and *pSyn1*-Cre::mCherry AAVs, calculated as the ratio of DAPI-stained cells with EGFP signal, from four confocal sections per SCN. (*C*) Representative traces of PER2::Luc bioluminescence from wild-type SCN slices before (black line) and after (colored line) transduction with AAVs encoding Cre::mCherry (*Top*) or tg-CRY1::EGFP, under minimal *pCry1* (*Middle*) or *pSyn1* (*Bottom*) promoters. (*C*, *Insets*) Three cycles before and four cycles after transduction reveal acute falls in baseline bioluminescence. (*D*) Group data (mean ± SEM) showing acute decrease in the trough of PER2::Luc bioluminescence following transduction of wild-type SCN with AAVs encoding *Cre::mCherry* (*Left*) or *tg-Cry1::EGFP*, under minimal *pCry1* (*Center*) or *pSyn1* (*Right*) promoters. (*E*) Group data (mean ± SEM) showing decrease of amplitude of PER2::Luc bioluminescence rhythm following transduction of wild-type SCN with AAVs encoding *Cre::mCherry* or *tg-Cry1::EGFP*, under minimal *pCry1* or *pSyn1* promoters. (*F* and *G*) Individual SCN data showing period (*F*) and goodness of fit (*G*) of wild-type SCN before and after transduction with AAVs encoding *Cre::mCherry* or *tg-Cry1::EGFP*, under minimal *pCry1* or *pSyn1* promoters. One SCN was excluded due to complete suppression of rhythmicity where no period could be determined. *pSyn1*-driven *tg-Cry1::EGFP* significantly lengthens period and worsens rhythms compared with the *Cre::mCherry* control or *pCry1*-driven *tg-Cry1::EGFP*.

If CRY1 is a state variable, the effect of expression of tg-CRY1 should vary depending on whether it is more or less rhythmic. To test this, circadian rhythms of PER2::Luc bioluminescence were recorded from wild-type SCN slices before and after transduction with a control AAV encoding Cre recombinase (with an mCherry tag) driven by *pSyn1* (AAV1-*pSyn1-Cre-mCherry*) or by the AAVs encoding tg-CRY1, either rhythmically (AAV1-*pCry1-Cry1::EGFP*) or constitutively (AAV1-*Syn1-Cry1::EGFP*). If constitutive, as opposed to circadian, expression of CRY1 is consistent with efficient TTFL function, both treatments should sustain well-organized rhythms. Post hoc, confocal microscopy of fixed slices (Fig. 2*A*) confirmed expression of tg-CRY1 via the EGFP tag, with no difference in transduction efficiency between the two vectors (Fig. 2*B*). The control *AAV-Cre-mCherry* had no effect on the ongoing oscillation of PER2::Luc bioluminescence (Fig. 2*C*). Rhythmically expressed tg-CRY1 had no significant effect on the baseline level of PER2::Luc bioluminescence, whereas constitutively expressed, *pSyn1*-driven tg-CRY1 caused an immediate and sustained (within two cycles) drop in baseline (acute normalized PER2::Luc trough: *pSyn1-Cre-mCherry*: 0.89 ± 0.01; *pCry1-Cry1*: 0.88 ± 0.01; *pSyn1-Cry1*: 0.66 ± 0.04; *P* = 0.001, one-way ANOVA) (Fig. 2*D*).

Treatment with control AAV or rhythmically expressed tg-CRY1 did not affect the normalized amplitude of the oscillation, whereas within two cycles of transduction with constitutively expressed tg-CRY1 there was a marked reduction (normalized acute treatment amplitude: *pSyn1-Cre-mCherry*: 0.88 ± 0.01; *pCry1-Cry1*: 0.84 ± 0.02; *pSyn1-Cry1*: 0.59 ± 0.06; *P* = 0.0005, one-way ANOVA) (Fig. 2*E*). The control AAV had no effect on the period of the oscillation, whereas rhythmically expressed tg-CRY1 caused a small lengthening (delta Period = 0.3 ± 0.1 h, *P* = 0.01, paired *t* test) (Fig. 2*F*). Constitutively expressed tg-CRY1, however, caused a greater lengthening of the ensemble period (delta Period = 2.3 ± 0.3 h, *P* = 0.009, paired *t* test) that was significantly greater than that seen with the *pCry1*-driven tg-CRY1 (*P* < 0.0001, unpaired *t* test) (note: One *pSyn1-Cry1* slice was excluded because the PER2::Luc bioluminescence was too damped to detect any oscillation).

The contrasting effects of rhythmic and constitutively expressed tg-CRY1 were also evident in a significant change in rhythmic goodness of fit (GOF), an inverse index of coherence [repeated-measures two-way ANOVA: AAV effect: *F*(2,14) = 13.62, *P* = 0.0005; pre/post effect: *F*(1,14) = 18.08, *P* = 0.0008; interaction effect: *F*(2,14) = 25.15, *P* < 0.0001] (Fig. 2*G*). Whereas SCN treated with control AAV or rhythmically expressed tg-CRY1 retained well-organized rhythms, there was a significant decline in the quality of rhythms of SCN constitutively expressing tg-CRY1 (Šidák’s multiple-comparisons test: *pSyn1-Cre-mCherry*, pre = 0.10 ± 0.01 vs. post = 0.13 ± 0.01, *P* = 0.79; *pCry1-Cry1*, pre = 0.13 ± 0.01 vs. post = 0.18 ± 0.01, *P* = 0.95; *pSyn1-Cry1*, pre = 0.13 ± 0.01 vs. post = 0.56 ± 0.1, *P* < 0.0001) (Fig. 2*G*). Thus, rhythmically expressed tg-CRY1 does not compromise the ongoing SCN oscillator. It allows robust, high-amplitude rhythms to persist, albeit with a slightly lengthened period. In contrast, constitutive expression of tg-CRY1 dramatically compromises the SCN oscillator, lengthening period, decreasing baseline, and rapidly damping and disorganizing the TTFL rhythm. These differential effects of rhythmic and constitutive expression of tg-CRY1 are consistent with CRY1 being a state variable of the TTFL of the SCN, but alone do not prove it.

### Translational Switching of Constitutive CRY1 Expression in the SCN.

The most informative tests of state-variable function in *Neurospora* and *Drosophila* exploited reversible transgenic induction of FRQ and Per, respectively. Importantly, this allows not only testing the actions of the protein of interest on the ongoing oscillation but also examining the effects of removing that transgenic protein from circulation within the TTFL. To achieve reversible, conditional control of tg-CRY1 expression, we used translational switching (*SI Appendix*, Fig. S1*A*), modifying our existing vector [AAV1-*pCry1-Cry1_(177TAG)_::EGFP*] (40) to support constitutive expression of translationally switched CRY1 (ts-CRY1) by replacing *pCry1* with the *pSyn1* promoter [AAV1-*pSyn1-Cry1_(177TAG)_::EGFP*] (*SI Appendix*, Fig. S1*B*). A second AAV expressed an orthogonal aminoacyl-tRNA (transfer RNA) synthetase/tRNA_CUA_ pair which enabled translational readthrough at the ectopic amber stop codon in the tg-*Cry1* messenger RNA (mRNA) to express ts-CRY1::EGFP, but only when the noncanonical amino acid alkyne-lysine (AlkK) was provided (41). Withdrawal of AlkK would curtail further CRY1_(177TAG)_::EGFP expression. The efficacy of the constructs was first confirmed by dual transfection of HEK293t cells. In the absence of AlkK, confocal imaging revealed expression of the mCherry tag on the synthetase but no expression of the C-terminal EGFP on ts-CRY1 (*SI Appendix*, Fig. S1*C*). The addition of AlkK triggered high levels of EGFP expression, indicative of ts-CRY1::EGFP expression. Western blots for EGFP confirmed the expression of the full-length fusion protein of appropriate molecular mass in cell extracts of HEK293t cells transfected with the nonswitchable *pSyn1*-*Cry1::EGFP* (*SI Appendix*, Fig. S1*D*). In cells transfected with synthetase and *pSyn1*-*Cry1_(177TAG)_::EGFP*, no ts-CRY1::EGFP was evident in the absence of AlkK, but the complete fusion protein was again present in cells treated with AlkK. The construct was then tested in SCN slices (*SI Appendix*, Fig. S1*E*). EGFP signal was not detected in SCN transduced with both AAVs but without AlkK, whereas 10 mM AlkK triggered strong EGFP signal indicative of ts-CRY1 expression. Native lysine did not support ts-CRY1::EGFP expression, confirming specificity of the AlkK (*SI Appendix*, Fig. S1*E*). To determine the time course of AlkK-induced expression, SCN transduced with the two AAVs for translational switching were imaged by confocal microscopy before and after addition of 10 mM AlkK. Before treatment, the mCherry tag on the synthetase was readily evident across the slice but there was no EGFP signal. Following addition of AlkK, strong EGFP signal emerged, indicative of ts-CRY1::EGFP expression (*SI Appendix*, Fig. S1*F*). Time-lapse imaging showed the accumulation of ts-CRY1::EGFP to be progressive, nonrhythmic, and linear for at least 10 d of treatment, consistent with the constitutive activity of the *Syn1* promoter. Following washout of the AlkK by repeated changes of culture medium, the ts-CRY1::EGFP signal disappeared rapidly. Translational switching thereby provided independent and reversible control of ts-CRY1 in a manner analogous to that used to control tg-FRQ expression using quinic acid induction (27).

### Translational Switching of CRY1 Expression Allows Reversible and Dose-Dependent Control of the Period and Amplitude of the TTFL of Wild-Type SCN.

**Fig. 3.**
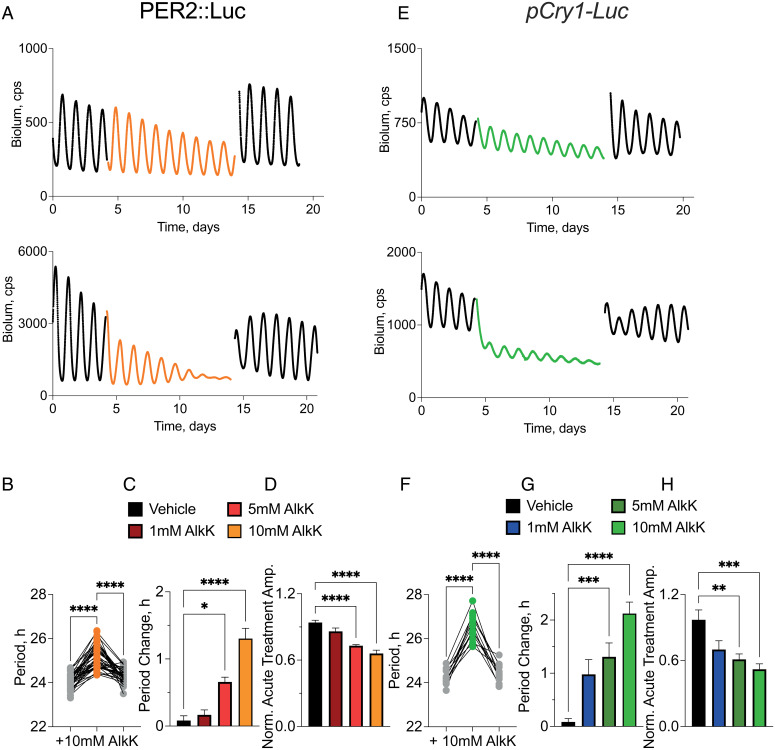
Reversible and dose-dependent control of TTFL period and amplitude of wild-type SCN by translationally switched CRY1::EGFP. (*A*) Representative traces of PER2::Luc bioluminescence from wild-type SCN slices transduced with AAVs to facilitate translational switching of constitutive (*pSyn1*-driven) CRY1::EGFP expression, before (black line) and during (colored line) treatment with vehicle (*Upper*) or 10 mM AlkK (*Lower*), and subsequent washout (black line). (*B*) Individual SCN data of the circadian period of PER2::Luc bioluminescence rhythms of SCN treated with 10 mM AlkK as in *A*, *Lower*. Period significantly and reversibly lengthens upon treatment with 10 mM AlkK. (*C* and *D*) Group data (mean ± SEM) showing (*C*) elongation of circadian period and (*D*) acute fall in amplitude of PER2::Luc bioluminescence rhythm of SCN treated as in *A* but with various doses of AlkK. Delta Period: vehicle: 0.1 ± 0.1 h, *n* = 11; 1 mM AlkK: 0.1 ± 0.1 h, *n* = 10; 5 mM AlkK: 0.7 ± 0.1 h, *n* = 10; 10 mM AlkK: 1.2 ± 0.1 h, *n* = 38; one-way ANOVA, *P* < 0.0001. Tukey’s multiple comparisons: vehicle vs. 1 mM: n.s.; vehicle vs. 5 mM: *P* = 0.0074; vehicle vs. 10 mM: *P* < 0.0001; 1 mM vs. 5 mM: *P* = 0.0288; 1 mM vs. 10 mM: *P* < 0.0001; 5 mM vs. 10 mM: *P* = 0.0097. Normalized posttreatment amplitude: vehicle: 0.92 ± 0.03; 1 mM AlkK: 0.86 ± 0.03; 5 mM AlkK: 0.71 ± 0.01; 10 mM AlkK: 0.66 ± 0.03; one-way ANOVA, *P* = 0.0002. Tukey’s multiple comparisons: vehicle vs. 1 mM: n.s.; vehicle vs. 5 mM: *P* = 0.17; vehicle vs. 10 mM: *P* < 0.0001; 1 mM vs. 5 mM: n.s.; 1 mM vs. 10 mM: n.s.; 5 mM vs. 10 mM: n.s. (*E*–*H*) As in *A* to *D*, but for SCN with *pCry1-Luc* reporter. (*E*) Representative bioluminescence traces. (*F*) SCN data showing reversible lengthening of period following treatment with and washout of 10 mM AlkK. (*G*) Group data (mean ± SEM) dose-dependent period change: vehicle: 0.1 ± 0.1 h, *n* = 13; 1 mM AlkK: 0.9 ± 0.1 h, *n* = 12; 5 mM AlkK: 1.4 ± 0.3 h, *n* = 11; 10 mM AlkK: 2.2 ± 0.2 h, *n* = 13; one-way ANOVA, *P* < 0.0001. Tukey’s multiple comparisons: vehicle vs. 1 mM: *P* = 0.027; vehicle vs. 5 mM: *P* = 0.0018; vehicle vs. 10 mM: *P* < 0.0001; 1 mM vs. 5 mM: n.s.; 1 mM vs. 10 mM: *P* = 0.0006; 5 mM vs. 10 mM: *P* = 0.0083. (*H*) Group data (mean ± SEM) showing acute fall in amplitude. Normalized posttreatment amplitude: vehicle: 0.88 ± 0.09; 1 mM AlkK: 0.70 ± 0.08; 5 mM AlkK: 0.61 ± 0.05; 10 mM AlkK: 0.54 ± 0.05; one-way ANOVA, *P* < 0.0001. Tukey’s multiple comparisons: vehicle vs. 1 mM: *P* = 0.0065; vehicle vs. 5 mM: *P* = 0.0002; vehicle vs. 10 mM: *P* < 0.0001; 1 mM vs. 5 mM: n.s.; 1 mM vs. 10 mM: n.s.; 5 mM vs. 10 mM: n.s.; *P < 0.05, **P < 0.01, ***P < 0.001, ****P < 0.0001 by Tukey’s multiple comparisons after ANOVA.

If CRY1 is a state variable, then progressively increased expression of ts-CRY1 should dose-dependently prolong the period of the TTFL by extending the interval of negative regulation and thereby delaying the reinitiation of CLOCK:BMAL1-dependent transcription. Second, with progressively higher levels of constitutive expression, the amplitude of the oscillation would be compromised. Finally, with sustained expression of ts-CRY1::EGFP, oscillation would be suspended as total CRY1 levels (endogenous plus ts-CRY1) were effectively clamped by the transgenic form. Transduction of wild-type SCN slices with AAVs encoding *pSyn1*-*Cry1_(177TAG)_::EGFP* and the synthetase had no effect on the circadian rhythm of PER2::Luciferase bioluminescence (Fig. 3*A*). Addition of vehicle was also without effect (*SI Appendix*, Fig. S2 *A* and *B*), whereas addition of 10 mM AlkK to trigger constitutive expression of ts-CRY1 resulted in significant changes. As with nonswitchable constitutive tg-CRY1, there was an acute drop in baseline bioluminescence (normalized treatment baseline: vehicle: 0.98 ± 0.09 of pretreatment; 10 mM AlkK: 0.68 ± 0.02; *P* = 0.0011, unpaired *t* test) (Fig. 3*A* and *SI Appendix*, Fig. S2*C*). The period of the PER2::Luc rhythm was significantly lengthened by 1.2 ± 0.1 h (*P* < 0.0001, *n* = 38, paired *t* test) and, importantly, this lengthening was completely reversed once AlkK was withdrawn by medium change (Fig. 3*B* and *SI Appendix*, Fig. S2*B*). Immediately after exposure to AlkK, the oscillation damped in amplitude (normalized acute treatment amplitude: vehicle: 0.92 ± 0.3; 10 mM AlkK: 0.66 ± 0.03; *P* < 0.0001, unpaired *t* test) (Fig. 3*D* and *SI Appendix*, Fig. S2*C*). The effects on period and amplitude were maintained until washout (*SI Appendix*, Fig. S2 *A* and *B*), and all of these effects of ts-CRY1—suppression of baseline, period lengthening, and amplitude damping—were dependent on the dose of AlkK (Fig. 3 *C* and *D* and *SI Appendix*, Fig. S2*C*). Serial recording of PER2::Luc bioluminescence and CRY1::EGFP fluorescence on representative SCN provided within-slice confirmation of the progressive rise in ts-CRY1 levels in parallel to the damping TTFL oscillation (*SI Appendix*, Fig. S1*F*). Moreover, at the highest dose of AlkK tested, 20 mM, oscillations of PER2::Luc were barely detectable before washout, with a mean final peak amplitude 3.5 ± 1.3% of the last peak prior to treatment and several slices showed oscillations with an amplitude less than 1% of the final peak before treatment (*SI Appendix*, Fig. S2*H*). This represents almost complete suppression of TTFL function.

To confirm that the effects of constitutive ts-CRY1 expression were general to the TTFL and not simply a specific action on the PER2::Luc reporter, we next tested the effect on SCN slices from transgenic reporter mice in which the minimal *Cry1* promoter drives luciferase expression (*pCry1-Luc*) (42). The *pCry1-Luc* reporter also provides a proxy for the effect of constitutive ts-CRY1 expression on the feedback-regulated, endogenous *Cry1* gene. Treatment with vehicle had no effect on the ongoing wild-type TTFL oscillation reported by *pCry1-Luc* (Fig. 3*E* and *SI Appendix*, Fig. S2 *D*–*F*), which had a slightly longer period (24.34 ± 0.07 h, *n* = 41) than the PER2::Luc SCN (23.90 ± 0.04 h, *n* = 99; *P* < 0.001, unpaired *t* test). Addition of 10 mM AlkK to trigger constitutive expression of ts-CRY1 caused a very rapid suppression of the baseline level of bioluminescence (normalized treatment baseline: vehicle: 0.95 ± 0.04%; 10 mM AlkK: 0.59 ± 0.02%; *P* < 0.0001, unpaired *t* test) (Fig. 3*E* and *SI Appendix*, Fig. S2*F*) and immediate damping (normalized acute treatment amplitude: vehicle: 0.88 ± 0.04; 10 mM AlkK: 0.54 ± 0.05; *P* < 0.0001, unpaired *t* test) (Fig. 3*H* and *SI Appendix*, Fig. S2*F*). Period was lengthened by 2.2 ± 0.2 h (*P* < 0.0001, paired *t* test) (Fig. 3*F* and *SI Appendix*, Fig. S2*E*) (note: Three slices were excluded from this analysis as the damping was so severe, no period could be determined), a greater response than observed in PER2::Luc SCN, but again the effects on period and amplitude were maintained until washout and the period lengthening was fully reversible (Fig. 3*G* and *SI Appendix*, Fig. S2 *E* and *F*). Finally, all of these effects were dose-dependent, reflecting the level of sustained ts-CRY1 expression (Fig. 3 *G* and *H* and *SI Appendix*, Fig. S2*F*). The results show that both translational (PER2::Luc) and transcriptional (*pCry1-Luc*) reporters of the circadian TTFL are sensitive to ts-CRY1, consistent with an effect on the TTFL independent of reporter type. They also confirm that translationally switched tg-CRY1::EGFP is able to determine TTFL period, amplitude, and baseline in a reversible, dose-dependent manner consistent with a role for CRY1 as a TTFL state variable.

### CRY1 Expression Regulates SCN Neuronal Activity.

**Fig. 4.**
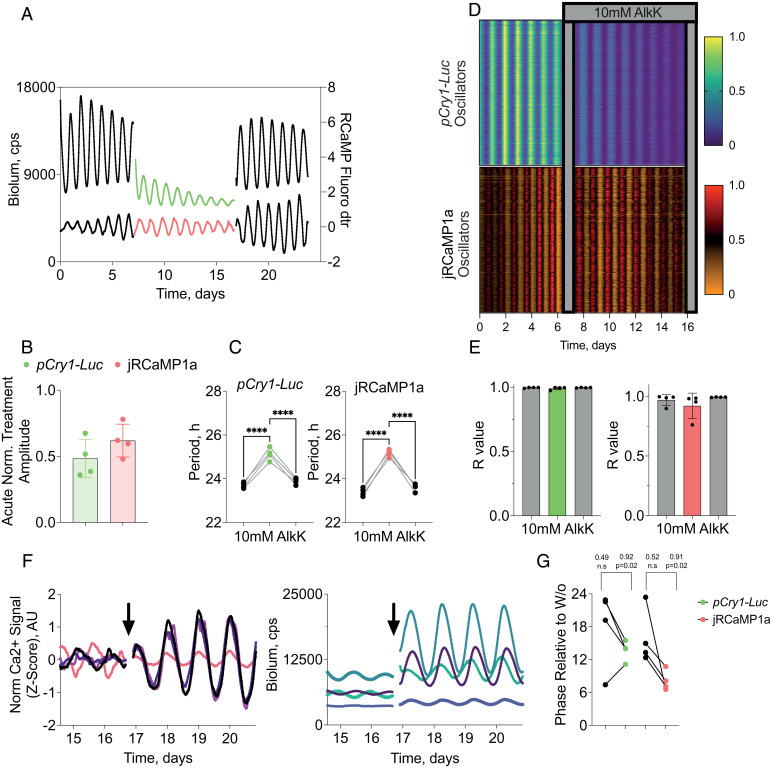
ts-CRY1::EGFP expression regulates SCN circadian neuronal activity. (*A*) Representative traces of *pCry1-Luc* bioluminescence (*Upper*; green) and neuronal [Ca^2+^]_i_ (*Lower*; red) from wild-type SCN slices transduced with AAVs to facilitate translational switching of constitutive (*pSyn1*-driven) CRY1::EGFP expression, before (black line) and during (colored line) treatment with 10 mM AlkK, and subsequent washout (black line). (*B*) Group data (mean ± SEM) of acute fall in amplitude of circadian *pCry1-Luc* and [Ca^2+^]_i_ rhythms of SCN as treated in *A*. Normalized to last cycle pretreatment. (*C*) Individual SCN data showing reversible elongation of period of circadian *pCry1-Luc* and [Ca^2+^]_i_ rhythms of SCN as treated in *A*. (*D*) Representative raster plots of cellular (region of interest) circadian rhythms of (*Upper*) *pCry1-Luc* and (*Lower*) [Ca^2+^]_i_ rhythms in a single SCN, before and during treatment with 10 mM AlkK as in *A*. Despite damping amplitude and lengthening period, constitutive CRY1::EGFP does not compromise intraslice synchrony. (*E*) Group data (mean ± SEM) showing intraslice cellular synchrony, as determined by Rayleigh vector before, during, and after treatment with 10 mM AlkK, in SCN slices, as in *A* (*Left*, *pCry1-Luc*; *Right*, [Ca^2+^]_I_; rhythms). (*F*) Traces of *pCry1-Luc* bioluminescence (*Left*) and neuronal [Ca^2+^]_i_ (*Right*) in SCN treated as in *A*, immediately before and for four cycles after washout (W/o) of 10 mM AlkK (arrows). AU, arbitrary unit. (*G*) Relative phases of *pCry1-Luc* bioluminescence (*Left*) and neuronal [Ca^2+^]_i_ (*Right*) in SCN treated as in *A*, immediately before (black dots) and for four cycles after (colored dots) washout of 10 mM AlkK. Values above denote Rayleigh synchrony estimates. *P < 0.05, **P < 0.01, ***P < 0.001, ****P < 0.0001 by Tukey’s multiple comparisons after ANOVA.

If CRY1 is a state variable, its manipulation would also be expected to affect TTFL-dependent, circadian-regulated aspects of SCN neuronal physiology and circuit-level organization. Intra-cellular calcium concentration ([Ca^2+^]_i_) is an index of neuronal activity that oscillates in SCN neurons with a peak at circadian time (CT) 08, in advance of the peaks of PER2 protein (CT12) and *Cry1* transcription (CT13.5) (43). This relationship was evident in charge-coupled device (CCD) dual recordings of bioluminescence and fluorescence from *pCry1-Luc* SCN transduced with AAV encoding the calcium reporter jRCaMP1a under the *pSyn1* promoter (Fig. 4*A*). The slices were also transduced with AAVs for translational switching and, consistent with photomultiplier tube data, treatment with 10 mM AlkK acutely damped the TTFL amplitude (normalized acute treatment amplitude = 0.48 ± 0.07) (Fig. 4*B*) and it reversibly lengthened the period of the bioluminescence rhythm (delta Period = 1.5 ± 0.1 h, *n* = 4, *P* = 0.0009, paired *t* test) (Fig. 4*C*). Neuronal activity rhythms, as reported by [Ca^2+^]_i_, were also sensitive to ts-CRY1 expression: Their amplitude damped to the same degree as did the *pCry1-Luc* oscillation (normalized acute treatment amplitude = 0.62 ± 0.06) and they also showed reversible period lengthening (delta Period = 1.8 ± 0.1 h, *P* = 0.0002, paired *t* test, *n* = 4) (Fig. 4 *B* and *C*). Thus, constitutive expression of ts-CRY1 acts on the rhythm of TTFL-dependent neuronal activity across the whole SCN.

**Fig. 5.**
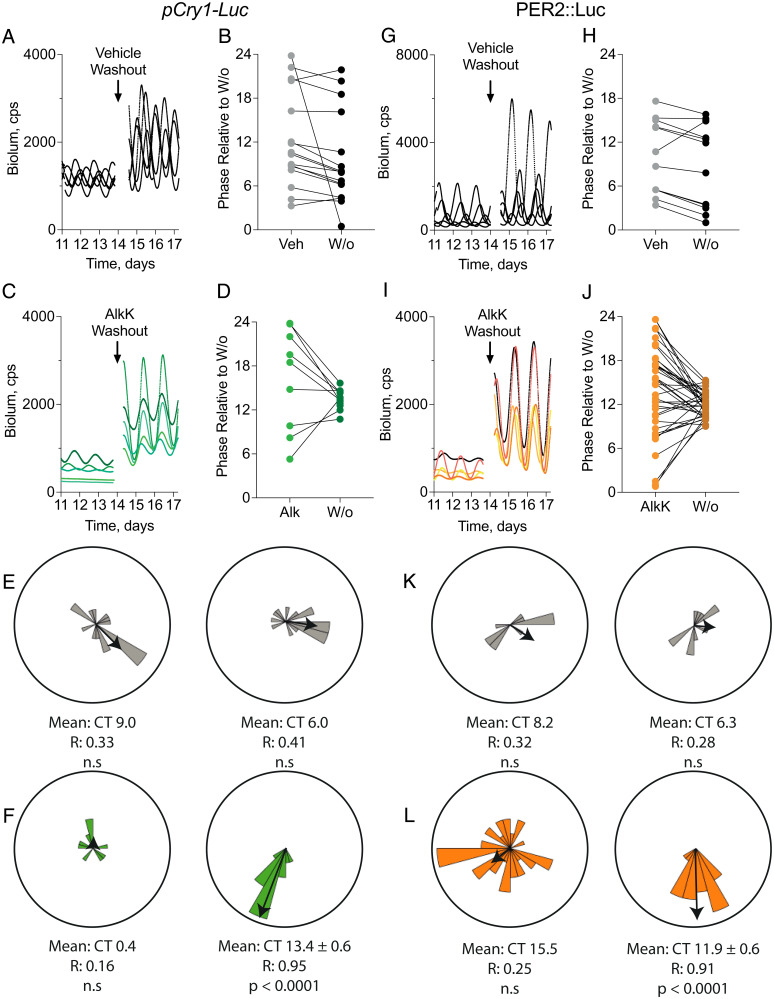
Acute withdrawal of ts-CRY1::EGFP resets SCN phase, realigning asynchronous slices to a common phase. (*A*) Representative *pCry1-Luc* bioluminescence traces from wild-type SCN, transduced with AAVs for translational switching of CRY1::EGFP expression, treated with vehicle, before and after washout (arrow). (*B*) Phases of individual SCN, treated as in *A*, before and after washout of vehicle expressed relative to time of washout. (*C*) Representative *pCry1-Luc* bioluminescence traces from wild-type SCN, transduced with AAVs for translational switching of CRY1::EGFP expression, treated with 10 mM AlkK, before and after washout (arrow). (*D*) Phases of individual SCN, treated as in *C*, before and after washout of 10 mM AlkK expressed relative to time of washout. (*E* and *F*) Rayleigh rose plots of SCN slices treated as in *A* and *C*, before (*Left*) and after (*Right*) washout of vehicle (*E*) and 10 mM AlkK (*F*). Rayleigh test of uniformity assesses the significance of the mean resultant length. The strongly significant result following 10 mM AlkK washout shows that the phases of independent SCN are no longer uniformly distributed and instead show strong synchrony around a mean resultant phase of CT13.4. (*G*–*L*) As in *A* to *F*, but for SCN with the PER2::Luc reporter. Independent SCN show synchrony after 10 mM AlkK washout, with a mean resultant phase of CT11.9. The reporter-specific phase confirms that at the point of AlkK withdrawal, both *pCry1-Luc* and PER2::Luc SCN restarted their oscillation from around CT0.

Damping of the ensemble signal could arise from loss of amplitude of cell-autonomous rhythms and/or desynchrony of the individual oscillators across the slice. CCD imaging of slices before treatment with AlkK confirmed the stereotypical intercellular synchrony, as reflected by raster pots (Fig. 4*D*) and Rayleigh analysis (Fig. 5*E*). Treatment with 10 mM AlkK and suppression of ensemble amplitude did not, however, affect phase coherence between neurons (Rayleigh vector length: *pCry1-Luc*: pre = 0.99 ± 0.01, AlkK = 0.99 ± 0.01, *P* = 0.40, one-way ANOVA; [Ca^2+^]_i_: pre = 0.97 ± 0.02, AlkK = 0.92 ± 0.05, *P* = 0.34, one-way ANOVA) (Fig. 4*E*). Consequently, washout of AlkK by medium change had no effect on neuronal synchrony. This demonstrates that while constitutive expression of ts-CRY1 reversibly damped the cell-autonomous TTFL of individual neurons, the electrical and neuropeptidergic networks across the SCN are still able to coordinate phase relationships between them. Such a cell-autonomous action of ts-CRY1 is consistent with a role as a state variable of the TTFL.

The reversibility of translational switching, allowing the imposition of a step change from elevated constitutive CRY1 levels back to endogenously regulated levels, offered a further opportunity to test this role. Following AlkK withdrawal, rhythms of *pCry1-Luc*–driven bioluminescence and [Ca^2+^]_i_ regained amplitude as damping by ts-CRY1 was terminated (Fig. 4*F*). Prior to washout, the different slices were oscillating independently, at low amplitude, and with no phase coherence (*pCry1-Luc*: pre washout, *R* = 0.41, *P* = 0.41; [Ca^2+^]_i_: pre washout, *R* = 0.53, *P* = 0.35). Following washout, the rhythms of the slices converged to a common, reporter-specific phase (*pCry1-Luc*: post washout, *R* = 0.92, *P* = 0.02, mean phase relative to washout = 13.7 ± 1.6 h; [Ca^2+^]_i_: post washout, *R* = 0.91, *P* = 0.02, mean phase relative to washout = 8.1 ± 1.5 h) (Fig. 4*G*). This suggested that on the step change from high to low levels of ts-CRY1, all slices adopted a common phase of oscillation. In limit cycles, acute changes to the level of a state variable propel the trajectory to a different phase, consistent with that new level. In the current context, the release from the elevated levels of the (transgenic) repressor CRY1 allowed the endogenous TTFL not only to recommence but also to do so by starting from the phase of elevated ts-CRY1–mediated repression.

### Phase Alignment after Withdrawal of ts-CRY1.

The common resetting following the step change in levels of CRY1 with all slices adopting a single defined phase is consistent with a role for CRY1 as a state variable of the SCN TTFL. To explore this further, we analyzed more extensively and precisely the consequences of AlkK withdrawal. AAV-transduced *pCry1-Luc* SCN were treated with vehicle or AlkK for 8 to 14 d, to damp their oscillation (Fig. 5 *A*, *C*, *G*, and *I*). Vehicle and AlkK were then withdrawn by medium change, without reference to the ongoing oscillation. Transgenic CRY1 translation following 10 mM AlkK severely damped oscillations but in most cases did not induce complete arrhythmicity (Fig. 3). This allowed assessment of phase before washout. The circadian phase of each slice was determined at the point of medium change, but because the slices had been cultured ex vivo for up to 1 mo before washout and were oscillating independently at their intrinsic period, there was no phase synchrony between different SCN at the point of washout (pretreatment phases relative to washout: vehicle: *R* = 0.33, not significant [n.s.]; AlkK: *R* = 0.16, n.s.). After medium change, the time taken for the bioluminescence to peak was calculated and compared within each treatment. The peaks of vehicle-treated SCN continued to be asynchronous after medium change (posttreatment phase: *R* = 0.41, n.s.) (Fig. 5 *B* and *E*). In contrast, after withdrawal of AlkK, the phases of the individual SCN became significantly synchronized (*R* = 0.95, *P* < 0.0001) (Fig. 5 *D* and *F*). A comparable phase-setting effect of AlkK withdrawal was observed in PER2::Luc SCN. Before washout, both vehicle- and AlkK-treated slices had no common phase (vehicle: *R* = 0.32, n.s.; AlkK: *R* = 0.25, n.s.) (Fig. 5 *G*, *I*, *K*, and *L*) and, after washout, vehicle-treated SCN remained asynchronous (*R* = 0.28, n.s.) (Fig. 5*K*). Following medium change in the asynchronous SCN treated with AlkK, however, the slices converged to a common phase and were highly synchronized (*R* = 0.91, *P* < 0.0001) (Fig. 5 *J* and *L*). The same significant alignment was seen in PER2::Luc SCN treated with a higher, 20 mM AlkK dose (before washout: *R* = 0.23, n.s.; after washout: *R* = 0.92, *P* = 0.0001; *n* = 17) (*SI Appendix*, Fig. S2*H*).

Importantly, the mean phase of the peak of *pCry1-Luc*–reported rhythms was 13.4 ± 0.6 h after washout, whereas the mean phase of the PER2::Luc-reported rhythms was 11.9 ± 0.6 h after washout. This phase difference between the two reporters is consistent with their relative phases within the free-running SCN TTFL cycle: Whereas *pCry1-Luc* rhythm peaks at CT13.5, PER2::Luc peaks at CT12.0 (43). By backextrapolation, therefore, the circadian phase of rhythms at the immediate point of AlkK withdrawal was CT0.1 (CT13.5 minus 13.4) according to the *pCry1-Luc* reporter, and CT0.1 (CT12 minus 11.9) according to the PER2::Luc reporter. After withdrawal of 20 mM AlkK, the reinitiated PER2::Luc rhythms peaked 11.5 ± 0.7 h after washout, corresponding to a phase at washout of CT23.5, consistent with the alignment seen at 10 mM. Given the variance in each group of SCN, these results are consistent with the asynchronous SCN being reset to ∼CT0 on withdrawal of AlkK (but not vehicle). This phase corresponds closely to the nadir of endogenous transcription of *Per* and *Cry* genes in the SCN, which occurs around CT0, and so a stepwise removal of constitutive CRY1 expression and reversion to normal, endogenous TTFL operation should cause the SCN to oscillate from this phase, regardless of its prewashout phase, as represented in the phase-transition plot (*SI Appendix*, Fig. S2*J*).

## Discussion

Virally mediated expression of tg-CRY1 induced TTFL rhythms in *Cry1/Cry2*-null SCN. We assume multiple signaling pathways coupled transduced (75 to 90%) and nontransduced cells because there was no obvious bimodality in population-level measures. If CRY1 is a state variable of the SCN clock, rather than a permissive auxiliary factor, three predictions follow (27): First, the efficacy of tg-CRY1 in driving the TTFL of arrhythmic *Cry1/Cry2*-null SCN should be determined by its temporal pattern of expression. Indeed, the effect varied with the timing of CRY1 expression. Mistimed rhythmic expression from the *pBmal1* promoter (44) could not sustain TTFL rhythms, whereas appropriately phased rhythmic expression from *pCry1* could (45). Expression from the *pSyn1* promoter, which this study verified is not rhythmic (*SI Appendix*, Fig. S1*F*), did initiate and sustain TTFL rhythmicity, a finding in agreement with the detection of rhythms in *Cry1/Cry2*-null fibroblasts treated with permeant CRY proteins (46) or transfected with plasmids constitutively expressing CRY1 from the CMV promoter (47). Nevertheless, the rhythms initiated in SCN by constitutively expressed tg-CRY1 were less stable than those induced by rhythmic and correctly phased tg-CRY1. Thus, the presence of tg-CRY1 at its correct phase is more important to TTFL function than its absence at other phases. Indeed, the endogenous oscillation of CRY1 protein sits completely above the threshold for interaction with PER2, and so it is never functionally absent (45). Overall, the timing of tg-CRY1 expression determines its effect on TTFL function, but the rhythms observed under *pSyn1-*driven tg-CRY1 mitigate against a simple model of a two-dimensional, CRY1-dependent limit cycle.

The second prediction is that constitutively elevated expression of tg-CRY1 in wild-type SCN should suppress the endogenous TTFL and extend its period. This was the case for *pSyn1-*driven tg-CRY1. In contrast, appropriately phased rhythmic expression of tg-CRY1 did not impair the wild-type TTFL. This is presumably because the rhythmic tg-CRY1 was able to engage with, and synchronize to, the endogenous TTFL, albeit lengthening the period slightly: a feature of the CRY1-dominated SCN (38, 44). The effect of sustained tg-CRY1 was independent of the reporter used, acting on both the *Per2* and *Cry1* axes, which is consistent with a fundamental role of tg-CRY1 within the TTFL. Indeed, the acute and sustained suppression of the transcription of *Cry1* by tg-CRY1 echoed the closed-loop effect of FRQ on the transcription of *frq* in the TTFL of *Neurospora* (27), further supporting a role for CRY1 as a state variable in the SCN.

The third and most critical prediction is that the level and direction of change of CRY1 expression must define the phase of the TTFL, and therefore an imposed change in level should shift the overt rhythm to a definitive phase. Reversible expression of tg-CRY1 by translational switching allowed us to perform this test. Provision of AlkK to express ts-CRY1 in wild-type SCN lengthened the period, suppressed the baseline, and damped the TTFL amplitude in the same way as did expression of nonswitchable tg-CRY1. Again, the effect was common to both PER2 and *Cry1* reporters and therefore general to the TTFL, although it should be noted that the PER2::Luc SCN were slightly less sensitive, possibly because the luciferase fusion affects PER2–CRY1 interactions. The suppression of neural activity by sustained expression of ts-CRY1 further emphasized its pervasive action on cell-autonomous oscillations. Significantly, network properties of the SCN were maintained in the presence of ts-CRY1, a feature also seen in SCN from mice carrying the *Fbxl3^Afh/Afh^* mutation which stabilizes CRY1 and similarly extends period (48). Thus, ts-CRY1 targeted the cell-autonomous TTFL of the SCN.

Suppression of the TTFL was dependent on the dose of AlkK and thus the level of ts-CRY1 expression (45). In all cases, the *pCry1-Luc* transcriptional reporter was affected more rapidly and strongly by elevated ts-CRY1 levels than was PER2 expression. This is likely because CRY1 directly suppresses transcription, which is reported as free luciferase by *pCry1-Luc*, whereas its effect on luciferase activity of the PER2 fusion reporter will be delayed and indirect, subject to posttranscriptional processes. At the highest doses (10 and 20 mM AlkK), the damping of TTFL amplitude was so severe that in some cases an oscillation could not be identified in the bioluminescence recordings, suggesting TTFL rhythmicity had been silenced. This condition of permanently elevated levels of ts-CRY1 associated with low and arrhythmic *Cry1* transcription and PER2 expression can be interpreted as a displacement of the steady-state TTFL trajectory, which was pushed to, and eventually locked at, a unique phase. This translocation was progressive as levels of ts-CRY1 increased linearly over the course of 10 d of treatment with AlkK. The reversibility of translational switching enabled the final test of phase determination. On removal of AlkK, ts-CRY1 disappeared (this study and ref. 40) and oscillation of the endogenous TTFL resumed. Backward extrapolation of the phase of the first subsequent peak confirmed that, regardless of the bioluminescent reporter used, the TTFL resumed from ∼CT0. This corresponds to the point of maximal CRY1-mediated transcriptional repression, with the TTFL in the “poised” state (49). Consequently, all individual SCN adopted a common phase in relation to washout, that is, were synchronized by the step change in levels of CRY1 (ts-CRY1 and endogenous CRY1) as they moved away from poised to active transcription (49). This meets the third criterion of a state variable.

The proposed CRY1-defined, steady-state limit cycle can be plotted (*SI Appendix*, Fig. S3*A*) as a phase-plane trajectory that registers *Cry1-Luc* bioluminescence (as a proxy for *Cry1* mRNA) against the oscillation of endogenous CRY1 protein, as monitored from SCN carrying a CRY1::mRuby knockin reporter (50). *Cry1* mRNA abundance peaks at CT13.5 (45) and CRY1 at CT18 (50) (*SI Appendix*, Fig. S3*A*). A similar plot can be made between *Cry1* mRNA and endogenous PER2 protein, again registered in circadian time (*SI Appendix*, Fig. S3*B*). During treatment with AlkK, *Cry1* mRNA declined rapidly. Although changes in endogenous CRY1 remain unknown, levels of PER2 can be used as a proxy for it, and so total CRY1 was estimated as endogenous PER2 plus the linear increase in ts-CRY1 observed directly. The proposed distortion and displacement of the limit cycle could then be inferred by plotting “total CRY1” against observed *Cry1-Luc* (*SI Appendix*, Fig. S3*C*). This placed the limit cycle at the high-CRY1, low-*Cry1* mRNA state, with a clamped oscillation. With washout of AlkK, ts-CRY1 levels declined rapidly, leaving total CRY1 at its endogenous nadir, as was *Cry1* mRNA, and so the limit cycle moved rapidly through phase space away from the “locked” point, first by increasing previously repressed transcription (*SI Appendix*, Fig. S1*D*). Subsequently, it reentered the steady-state trajectory as endogenous CRY1 and PER2 were expressed with progressively larger amplitude oscillations (again, endogenous PER2 was used as a proxy for endogenous CRY1 in the washout condition).

Notwithstanding the incomplete and semiquantitative nature of this interpretation of the experimental results, the inferred trajectories provide a framework to explore further the properties of the SCN TTFL. In terms of molecular composition, the mammalian TTFL is more complex than that of *Neurospora* and so it is likely that several factors beyond CRY1 act as state variables in the SCN and other mammalian clock cells. CRY2 is a weaker negative regulator and may function principally to “tune” the effect of CRY1 (38). Moreover, short-period (∼16-h) circadian cycles of PER2::Luc expression are transiently expressed by *Cry1/Cry2*-null neonatal SCN slices (33, 51, 52), indicative of additional CRY-independent oscillatory mechanisms that may be posttranslational and/or cytosolic in nature (33, 53). Furthermore, because *Per1/Per2*-null mice and SCN are arrhythmic (54) and resetting SCN phase is accompanied by changes in *Per* transcription (5), autoregulation within the *Per1* and *Per2* axes may allow PER1 and/or PER2 to be state variables of the SCN TTFL. The observation that weak, poorly defined oscillations, with periods well outside the circadian range, can be seen in the absence of CRY proteins (33) does not, however, undermine the positive evidence for CRY1 as a state variable provided by translational switching. Indeed, we should note the value of translational switching, achieved by genetic code expansion, for reversible, dose-dependent, and conditional control over the expression of a protein of interest. By their nature, the current studies demonstrate how readily neurons in culture tolerate for many weeks the expression of orthogonal tRNA synthetase, tRNA, and amber suppression to mediate translational readthrough. The operation of the SCN TTFL is so precise and robust that any off-target or metabolic compromise caused by these manipulations would have been readily apparent. The absence of such effects during repeated cycles of treatment emphasizes the nontoxic and selective nature of the approach. More generally, reversible dose-dependent control over the expression of a protein of interest may have broad utility in biomedical applications. In a circadian context, translational switching may therefore be useful to determine the potential roles of PER1 and PER2 as state variables, and thereby construct a multidimensional limit-cycle model of the SCN TTFL.

## Materials and Methods

A detailed description of materials and methods is provided in *SI Appendix*. Itemized lists contain the provenance of all animals and previously generated AAVs used. Descriptions of SCN organotypic slice preparation and all subsequent SCN recording procedures (confocal live/fixed imaging, LV200 microscope system imaging, and luciferase recordings) are also included. Methodology of all analyses (circadian and image-based) is summarized. The R package circular was used to perform circular statistics on phase data, and we provide a list of which built-in functions were used.

## Supplementary Material

Supplementary File

## Data Availability

An Excel spreadsheet of bioluminescence recording data has been deposited in BioDare2, https://biodare2.ed.ac.uk/experiment/ (55–73) and the relevant experimental IDs can be found in *SI Appendix*, Table S3. Biodare access is not password-protected. All study data are included in the article and/or *SI Appendix*.
