# Supplementary material for: Cryptochrome 1 as a state variable of the circadian clockwork of the suprachiasmatic nucleus: Evidence from translational switching
Source: Proc Natl Acad Sci U S A. 2022 Aug 17;119(34):e2203563119. doi: 10.1073/pnas.2203563119 (PMC9407638; doi:10.1073/pnas.2203563119)
Supplement: Supplementary File [file pnas.2203563119.sapp.pdf]

Supporting Information for: Cryptochrome 1 as a state-variable of the circadian clockwork of the suprachiasmatic nucleus: evidence from translational switching.

David McManus, Lenka Polidarova, Nicola J. Smyllie, Andrew P. Patton, Johanna E. Chesham, Elizabeth S. Maywood, Jason W. Chin, Michael H. Hastings

Corresponding author: Michael H. Hastings
Email: mha@mrc-lmb.cam.ac.uk

This PDF file includes:

Supporting text
Figures S1 to S3
Tables S1 to S3
SI References

Supporting Information: Materials

Animals

All animal work was conducted under UK Home Office licence and overseen by the Animal Welfare and Ethical Review Body of the MRC Laboratory of Molecular Biology, under Animals (Scientific Procedures) Act of 1986. Animals were maintained on a 12:12 light-dark schedule. Animal provenance is set out in Table S1.

AAV

AAV design and production details are set out in Table S2

Supporting Information: Methods

Organotypic SCN slice preparation

SCN slices were prepared as previously described (9) and outlined briefly below. Both male and female mouse pups aged between P9-P12 were culled by a Schedule 1 method (dislocation and exsanguination) and brain removed, which was then quickly transferred to ice-cold GBSS (Sigma, USA) dissection medium (5 mg/mL glucose, 100 nM MK801 (Sigma, USA); 3mM MgCl2 and 0.05 mM AP5, (Sigma); to block excitotoxicity). After trimming, 300 µm coronal brain slices were prepared on a McIlwain (U.K.) tissue chopper. The slice(s) containing the SCN was/were trimmed down further, minimising any extraneous non-SCN tissue. The SCN slices were transferred to membrane inserts (Milipore, USA) sitting on top of tissue culture medium (50% Eagle's basal medium (Sigma); 25% ESS (Sigma); 25% heat inactivated horse serum (Invitrogen, USA); 5 mg/mL D-glucose (Sigma); 25 µg/mL Penicillin/Streptomycin; 1% Glutamax (Invitrogen); pH7.2, osmolarity 315-320 mOsm), supplemented with 100 nM MK801 (Thermo Fisher Scientific, USA), 3mM MgCl2 and 0.05 mM D-AP5 (Tocris, USA), to block excitotoxicity. After 2-4 hours, slices were transferred to fresh culture medium, without the excitotoxicity blockers, in 6-well dishes for long-term culturing (maintained at 37°C, 5% CO2). SCN slices were cultured prior to experiments involving luciferase recordings, or transferred directly to recording medium (see below) for experiments involving live fluorescence imaging.

Luciferase recordings

SCN slices were transferred to 35 mm dishes containing 1.2 mL recording medium (D-MEM (Sigma); 0.35 mg/mL NaHCO3 (Fisher Scientific); 5 mg/mL glucose; 25 µg/mL Penicillin/Streptomycin; 0.01M HEPES (Invitrogen)) made up as a stock solution and further supplemented with foetal calf serum (Gibco, USA), B27 (Gibco), Glutamax (Invitrogen) and 100 µM Luciferin (Microsynth, Switzerland)). Dishes were sealed with a cover-glass secured using silicone grease to prevent evaporation of media during the recording. Luciferase bioluminescence was

detected by a photon multiplier tube (PMT; Hamamatsu, Japan), maintained at 37 °C in a light-tight incubator (CO2 not required as slices were sealed and buffered with HEPES and NaHCO3). Photon counts were recorded every second and counts were combined in 6-minute bins.

Viral transduction of SCN slices

SCN slices were transduced with AAVs after a medium change (culture medium or recording medium, depending on the experiment) immediately prior to transduction. One µL of AAV with titre of at least 1x1013 GC/mL was dispensed directly on top of the SCN slice and incubated for 7 days before exchanging for fresh medium.

Translational switching

SCN slices were sequentially incubated with each AAV for 7 days separated by a medium change with fresh culture medium. To allow translation of tgCRY1(TAG) the substrate for PylRS, alkene lysine, abbreviated to AlkK, N6-2-propynyloxycarbonyl-l-lysine, (synthesised in-house, stored as 100 mM stock solution made in recording medium, adjusted to pH 7.0) was added to the recording medium with a final concentration of 0.1 - 10 mM. Reversibility was confirmed by exchanging fresh recording medium that did not contain AlkK, using 6 washouts for 15 minutes each. In the dose response experiments, AlkK was applied to give a final concentration of either 0, 1, 5 or 10 mM to the double transduced SCN slices.

Confocal time-lapse recordings

Live imaging of SCN organotypic slices was carried out using either Zeiss LSM780 or LSM880 inverted confocal systems, maintained at 37°C. Custom inserts were made to hold up to 6x 35 mm dishes at a time. The “position list” function was used to make simultaneous time-lapse recordings from multiple SCN slices. SCN slices were transferred to 35 mm glass bottom dishes (Mattek) for all live imaging experiments. The same recording medium was used as for the PMT luciferase recordings. For time-lapse recordings, a 10X apochromatic objective was used with the following acquisition parameters: 1024 x 1024 pixel frame size, 4x averaging, 1 frame acquired every 30 minutes for the duration of the experiment.

SCN fixed slices

SCN slices were cut out of their membrane inserts and fixed in 4% PFA in phosphate buffer (as previously) for 30 minutes at room temperature, with gentle shaking. Fixed SCN slices were washed with PBS for 15 minutes, 3 times and mounted on to slides (as previously) with mounting medium with DAPI.

Confocal snapshot imaging

Fixed SCN tissue (sections and slices) were imaged using either Zeiss LSM710, 780 or 880 systems using a 63x oil immersion apochromatic objective. To image the whole coronal view of the SCN, a tile-scan protocol was used within the Zen acquisition software. Particularly in the DAPI channel, this produced some tile artifacts at the joins between tiles, which can be seen in images. Nonetheless, the tile-scan allowed for higher resolution images of the whole SCN than could be acquired with the highest numerical aperture (NA) 10x objective, an objective that would not have required tiling. Further processing was carried out within FIJI (10).

BioDare

The Fast Fourier Transform – Linear Non-Least Squares (FFT-NLLS) within the BioDare (www.biodare2.ed.ac.uk) circadian rhythms analysis software package (11) was used to analyse rhythmicity of both luciferase and fluorescence-based recordings, where there was stable rhythmicity of at least 5 days. The first 24 hours after any medium change or treatment were excluded from the analysis. The output from software included best-fit period within a circadian-relevant window of 18-32 h periods, relative peak phase, amplitude, goodness of fit (GOF), the latter is an assessment of the robustness of the rhythms. Shapiro-Wilk test of our GOF dataset, in particular the GOF measure of oscillations from all SCN before AAV transduction, (Figure 2G) showed it to be normally distributed, $p = 0.89$. An ANOVA is therefore an appropriate test to use when making comparisons between these data.

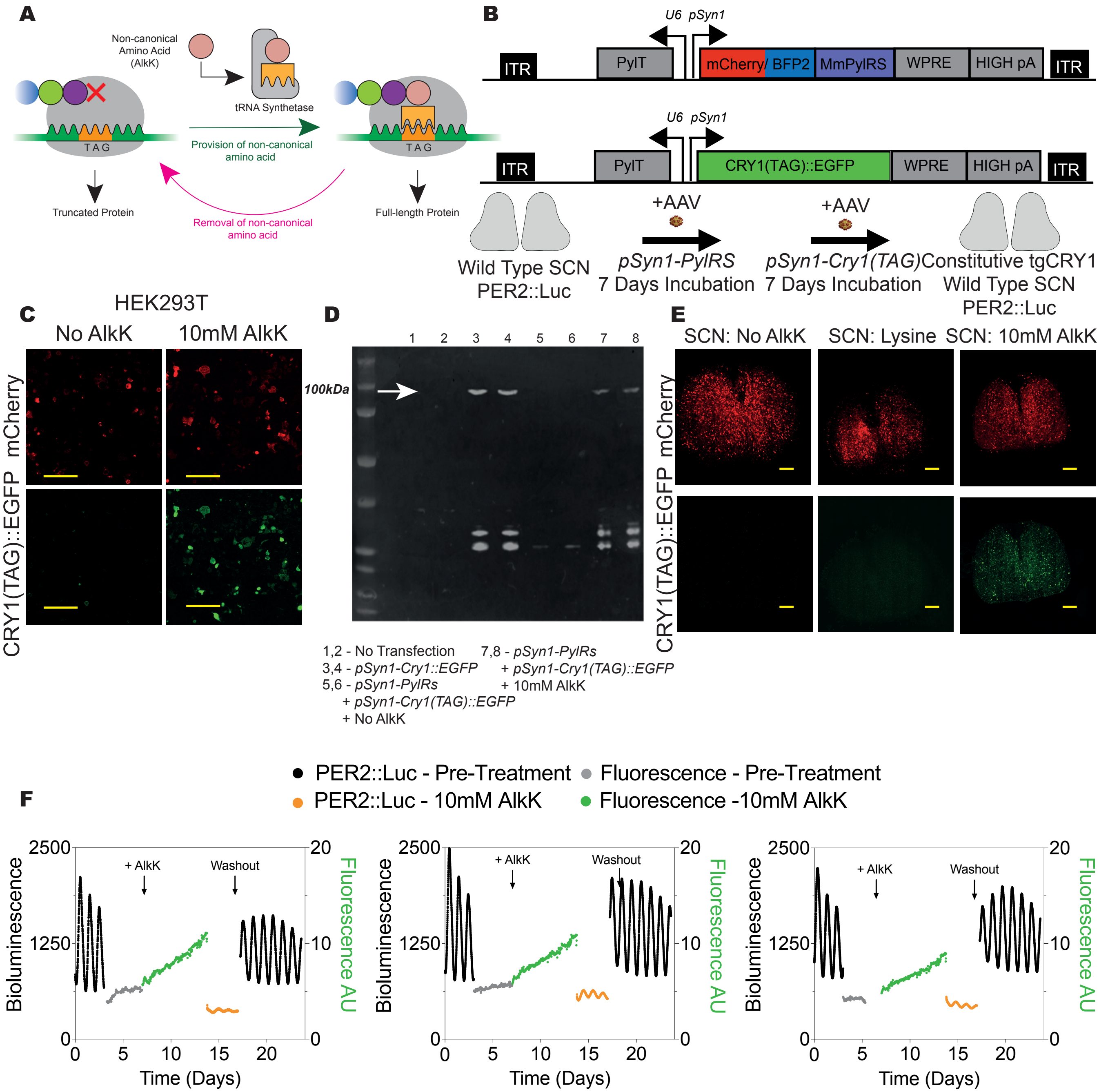

A
Non-canonical Amino Acid (AlkK)
tRNA Synthetase
TAG
Truncated Protein
Provision of non-canonical amino acid
Removal of non-canonical amino acid
Full-length Protein
B
U6 pSyn1
ITR
PylT
mCherry/ BFP2
MmPylRS
WPRE
HIGH pA
CRY1(TAG)::EGFP
+AAV
Wild Type SCN PER2::Luc
pSyn1-PylRS 7 Days Incubation
pSyn1-Cry1(TAG) 7 Days Incubation
Constitutive tgCRY1 Wild Type SCN PER2::Luc
C
HEK293T
No AlkK
10mM AlkK
mCherry
CRY1(TAG)::EGFP
D
1 2 3 4 5 6 7 8
100kDa
1,2 - No Transfection
3,4 - pSyn1-Cry1::EGFP
5,6 - pSyn1-PylRs + pSyn1-Cry1(TAG)::EGFP + No AlkK
7,8 - pSyn1-PylRs + pSyn1-Cry1(TAG)::EGFP + 10mM AlkK
E
SCN: No AlkK
SCN: Lysine
SCN: 10mM AlkK
F
PER2::Luc - Pre-Treatment
Fluorescence - Pre-Treatment
PER2::Luc - 10mM AlkK
Fluorescence -10mM AlkK
Bioluminescence
Fluorescence AU
+ AlkK
Washout
Time (Days)

Fig. S1. Validation of translational switching to control CRY1::EGFP expression in wild-type SCN.

A. Schematic of translational switching approach, redrawn from (45).

B. Constructs used for translational switching of CRY1::EGFP expression (upper) and general experimental sequence (lower), redrawn from (45).

C. Representative images of Hek293t cells, transfected with plasmids for translational switching of CRY1::EGFP, untreated (left) or treated with 10 mM AlkK (right). Red channel (upper panels) shows expression of mCherry tag on tRNA synthetase, green channel (lower) shows ts-CRY1::EGFP.

D. Western blot confirms expression in Hek293t cells of full length non-switched CRY1::EGFP (lanes 3, 4) and by ts-CRY1::EGFP (arrowed), but only in presence of 10 mM AlkK (lanes 7, 8).

E. Representative images of SCN slices transduced with AAVs for translational switching, before (left) and after (right) treatment with 10 mM AlkK to induce ts-CRY1::EGFP expression, or treated with unmodified lysine as a negative control (centre). Red channel (upper panels) shows expression of mCherry tag on tRNA synthetase, green channel (lower) shows ts-CRY1::EGFP.

F. Representative recordings of the intensity of ts-CRY1::EGFP signal in PER2::Luc SCN slices transduced with AAVs for translational switching. Bioluminescence was first recorded from SCN (black, left) in PMTs. SCN were then moved to a confocal microscope where ts-CRY1::EGFP signal was recorded before (grey) and during (green) 10mM AlkK treatment. After 7 days of AlkK treatment, SCN were transferred back to PMTs to record suppressed bioluminescence during ts-CRY1 expression (orange). AlkK was then washed out via medium change and PER2::Luc bioluminescence was recorded post removal of ts-CRY1::EGFP (black, right).

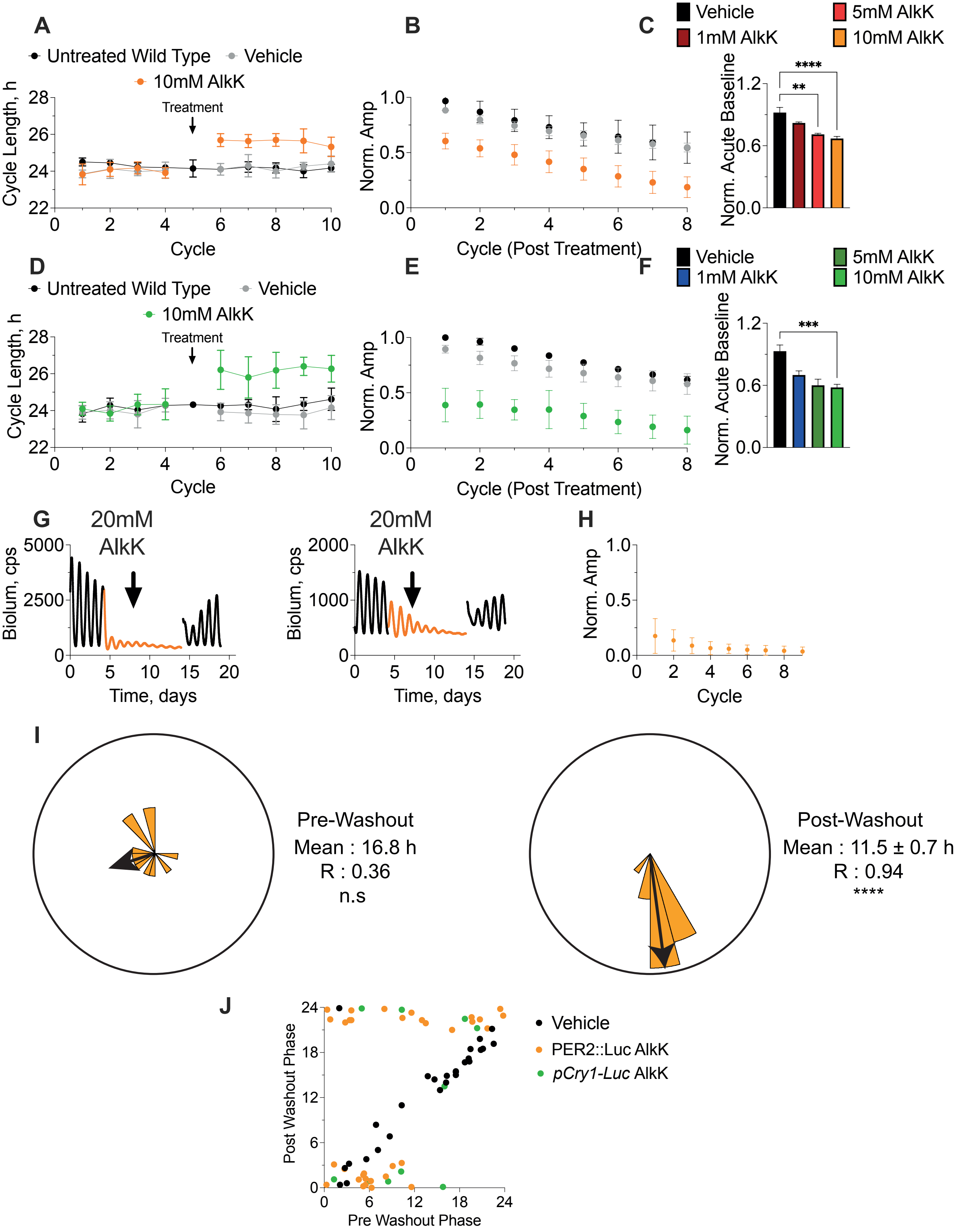
A
Untreated Wild Type
Vehicle
10mM AlkK
Treatment
Cycle Length, h
Cycle
B
Norm. Amp
Cycle (Post Treatment)
C
Vehicle
1mM AlkK
5mM AlkK
10mM AlkK
Norm. Acute Baseline
****
**
D
Untreated Wild Type
Vehicle
10mM AlkK
Treatment
Cycle Length, h
Cycle
E
Norm. Amp
Cycle (Post Treatment)
F
Vehicle
1mM AlkK
5mM AlkK
10mM AlkK
Norm. Acute Baseline
***
G
20mM AlkK
Biolum, cps
Time, days
20mM AlkK
Biolum, cps
Time, days
H
Norm. Amp
Cycle
I
Pre-Washout
Mean : 16.8 h
R : 0.36
n.s
Post-Washout
Mean : 11.5 ± 0.7 h
R : 0.94
****
J
Post Washout Phase
Pre Washout Phase
Vehicle
PER2::Luc AlkK
pCry1-Luc AlkK

Fig. S2. Reversible and dose-dependent control of TTFL period, amplitude and phase of wild-type SCN by translationally switched CRY1::EGFP.

A. Mean (+SEM) cohort cycle length of wild type PER2::Luc SCN transduced with AAVs to facilitate translational switching (ts) of constitutive (pSyn1-driven) CRY1::EGFP expression before and during treatment (black arrow) with vehicle (grey) or 10mM AlkK (orange). Cycle lengths from untreated PER2::Luc bioluminescence are presented (black) as a comparison.

B. Mean (+SEM) cohort cycle-to-cycle amplitude of wild type PER2::Luc SCN transduced with AAVs to facilitate constitutive (pSyn1-driven) ts-CRY1::EGFP expression during treatment with vehicle (grey) or 10mM AlkK (orange). Cycle-to-Cycle amplitude from untreated PER2::Luc bioluminescence is presented (black) as a comparison. Amplitude calculated for each cycle during treatment was normalised to the amplitude of the peak pre-treatment.

C. Group data (Mean +SEM) showing acute fall in baseline of wild type PER2::Luc bioluminescence rhythm of SCN transduced with AAVs to facilitate expression of constitutive pSyn1-driven, ts-CRY1::EGFP expression treated with various doses of AlkK. Acute treatment baseline, Vehicle: 0.98±0.05, 1mM AlkK: 0.82±0.01, 5mM AlkK: 0.73±0.01, 10mM AlkK: 0.68±0.02, one way ANOVA $p<0.0001$. Tukey's multiple comparisons, Vehicle vs 1mM: n.s., Vehicle vs 5mM: $p = 0.005$, Vehicle vs 10mM: $p<0.0001$, 1mM vs 5mM: n.s., 1mM vs 10mM: $p = 0.01$, 5mM vs 10mM: n.s.

D. As with A but for wild type pCry1-Luc SCN. Vehicle (grey), 10mM AlkK (green) and, untreated wild type pCry1-Luc SCN (black).

E. As with B but for wild type pCry1-Luc SCN. Vehicle (grey), 10mM AlkK (green) and untreated wild type pCry1-Luc SCN (black).

F. As with C, but for wild type pCry1-Luc SCN treated with various doses of AlkK. Acute treatment baseline, Vehicle: 0.95 ±0.04, 1mM AlkK: 0.70 ±0.04, 5mM AlkK: 0.60 ±0.05, 10mM AlkK: 0.59 ±0.02, one way ANOVA $p<0.0001$. Tukey's multiple comparisons, Vehicle vs 1mM: n.s., Vehicle vs 5mM: $p = 0.0002$, Vehicle vs 10mM: $p<0.0001$, 1mM vs 5mM: n.s., 1mM vs 10mM: n.s., 5mM vs 10mM: n.s.

G. Representative traces of PER2::Luc bioluminescence from wild-type SCN slices transduced with AAVs to facilitate constitutive, pSyn1-driven ts-CRY1::EGFP expression, before (black line) and during (colored line) treatment with 20 mM AlkK, and subsequent washout (black line).

H. Mean (+SEM) cohort cycle-to-cycle amplitude of wild type PER2::Luc SCN transduced with AAVs to facilitate constitutive (pSyn1-driven) ts-CRY1::EGFP expression during treatment with 20mM AlkK. Amplitude calculated for each cycle during treatment was normalised to the amplitude of the last peak pre-treatment.

I. Rayleigh rose plots of SCN slices treated as in H, before (left) and after (right) washout of 20 mM AlkK.

* <0.05, ** p<0.01, *** p<0.001, **** p<0.0001 by Tukey's multiple comparisons test after ANOVA. J. Phase-transition curve of both PER2::Luc SCN and pCry1-Luc SCN following washout of vehicle (black) or 10mM AlkK (colored) treatments. Phase is determined as the phase of the oscillation at the point of washout/ immediately post washout. Washout of AlkK shows type 0 resetting, as oscillations all restart from ~CT0 regardless of the phase before.

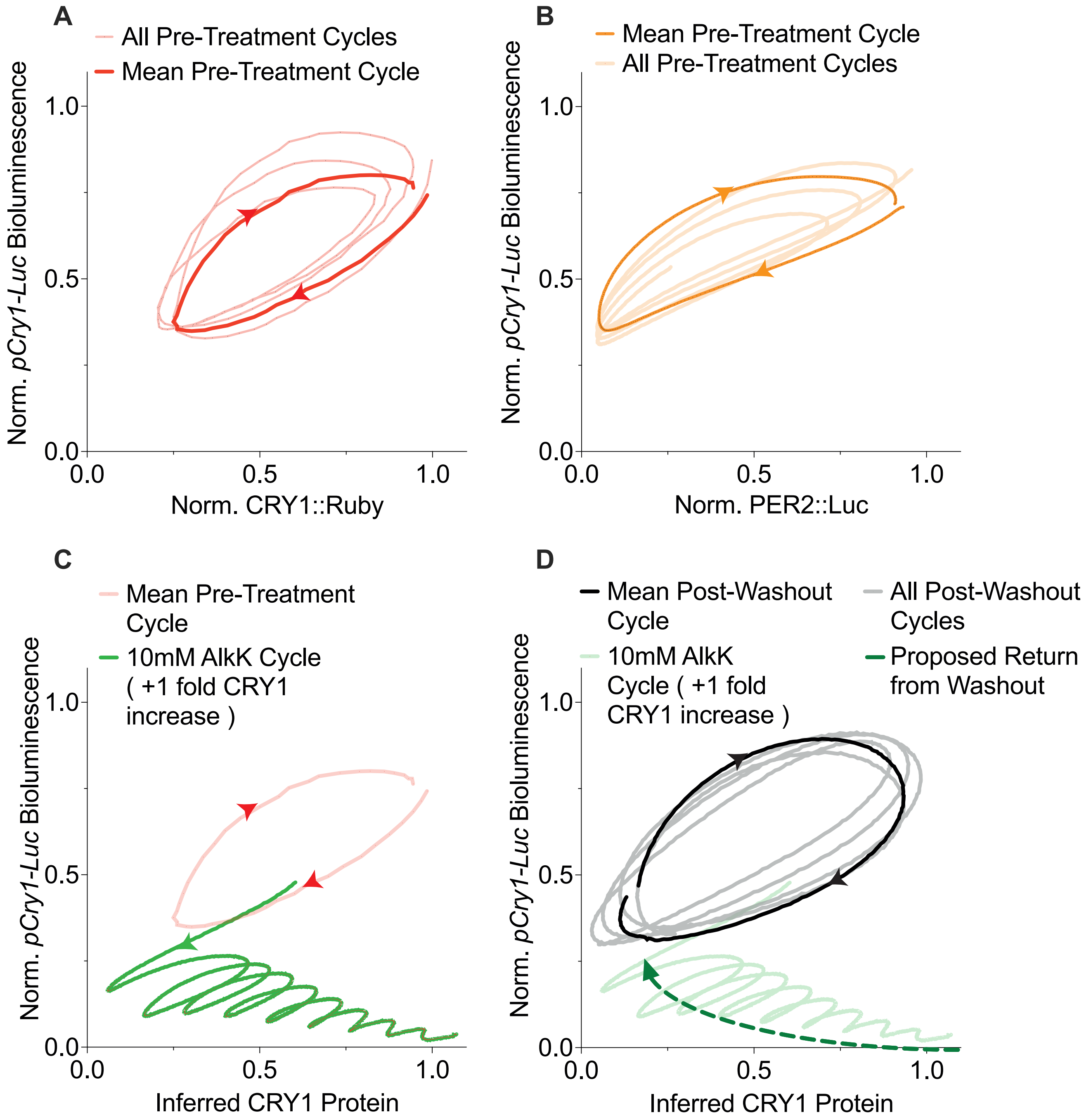
A
All Pre-Treatment Cycles
Mean Pre-Treatment Cycle
Norm. pCry1-Luc Bioluminescence
Norm. CRY1::Ruby
B
Mean Pre-Treatment Cycle
All Pre-Treatment Cycles
Norm. pCry1-Luc Bioluminescence
Norm. PER2::Luc
C
Mean Pre-Treatment Cycle
10mM AlkK Cycle (+1 fold CRY1 increase)
Norm. pCry1-Luc Bioluminescence
Inferred CRY1 Protein
D
Mean Post-Washout Cycle
All Post-Washout Cycles
10mM AlkK Cycle (+1 fold CRY1 increase)
Proposed Return from Washout
Norm. pCry1-Luc Bioluminescence
Inferred CRY1 Protein
0.0
0.5
1.0

Figure S3. Representation of inferred 2-dimensional limit cycle trajectories as phase-plane diagrams

A. Phase-plane diagram created by plotting the normalised mean of pCry1-Luc oscillations recorded from five individual SCN against the normalised mean of CRY1::Ruby oscillations from five other SCN, cross-registered in circadian time. This figure re-plots data regarding circadian CRY1 protein abundance from Koch et al., (2022), eLife PMID: 35285799, which was published under the Creative Commons Attribution License (CC-BY 4.0):
https://creativecommons.org/licenses/by/4.0/.
No changes to the data were made. Mean of five cycles (opaque red) and individual cycles (translucent red).

B. Phase-plane diagram plotted as in A, but with PER2::Luc bioluminescence recordings from five SCN in place of CRY1::Ruby fluorescence. Mean of five cycles (opaque orange) and individual cycles (translucent orange). Note comparability to A.

C. Phase-plane diagram showing inferred limit cycle trajectory during treatment with AlkK. Level of CRY1 is estimated by using endogenous PER2 data as a proxy for endogenous CRY1, to which is added a linear increase arising from ts-CRY1. Expression of ts-CRY1 causes acute falls in estimated CRY1 and Cry1 mRNA. During 10 days of 10mM AlkK treatment the oscillation damps as CRY1 rises progressively to its final maximal level (green). At this point the limit cycle is locked into a high CRY1 protein, low Cry1 mRNA state. Translucent plot is pre-treatment trajectory, as in A.

D. Inferred behavior of limit cycle on withdrawal of ts-CRY1. Confocal imaging showed that signal from ts-CRY1::EGFP was lost within 12 hours of wash-out. Removal of AlkK was also followed by an increase in Cry1 mRNA, which is represented by the dotted trajectory, moving away from the locked state. All SCN re-commenced full amplitude oscillations from the putative CT00. The individual four cycles starting 24 hours after wash-out (mean of five SCN) are plotted in grey, and their overall mean in black, again using PER2 as a proxy for endogenous CRY1.

Table S1. Animal Provenance

#	Strain name	Alleles	Provenance	Ref.
1	CryDKO (*Cry1/Cry2*-null)	*mCry1*$^{-/-}$;*mCry2*$^{-/-}$	Gijsbertus van der Horst, Erasmus Univ.	(1)
2	PER2::Luc	*mPer2*$^{Luc/Luc}$	Joseph Takahashi, UTSW	(2)
3	*Cry1-Luc*	*pCry1-Luc*	In house at MRC-LMB	(3)

Table S2. AAV design and production details

<table>
<tr><td>AAV full name</td><td>Construct provenance</td><td>AAV Packaging</td></tr>
<tr><td>AAV.pCry1.Cry1::EGFP</td><td rowspan="3">Modified in house from (4)</td><td rowspan="6">Penn Vector Core (University of Pennsylvania, USA)</td></tr>
<tr><td>AAV.pSyn1.Cry1::EGFP</td></tr>
<tr><td>AAV.pBmal1.Cry1::EGFP</td></tr>
<tr><td>AAV.pSyn1.mCherry.P2A.PylRS</td><td rowspan="2">In house (5) (6)</td></tr>
<tr><td>AAV.pSyn1.BFP2.P2A.PylRS</td></tr>
<tr><td>AAV.pSyn1.Cry1(177TAG)::EGFP</td><td>Modified in house from (7)</td></tr>
<tr><td>AAV.Syn.NES.jRCaMP1a</td><td>Addgene viral prep # 100848-AAV1 https://www.addgene.org/100848/</td><td>Addgene</td></tr>
<tr><td>AAV.pSyn1.Cre.mCherry</td><td>AAV-hSyn-mCherry-P2A-Cre-WPRE was a gift from Hui Yang (Addgene plasmid #107312; http://n2t.net/addgene:107312; RRID:Addgene_107312) (8)</td><td>Addgene</td></tr>
</table>

Table S3. Experimental IDs for all datasets, available at BioDare repository https://biodare2.ed.ac.uk/experiment/nnnnn

	Data	Experiment ID
Figure 1	CryDKO + pBmal.Cry1	22521
	CDKO + pCry1.Cry1	22520
	CDKO + pSyn1.Cry1	22519
Figure 2	WT + Cre.mCherry (Control)	22516
	WT + pCry1.Cry1	22514
	WT + pSyn1.Cry1	22515
Figure 3	PER2::LUC + pSyn1.Cry1(TAG)::EGFP, Vehicle	22836
	PER2::LUC + pSyn1.Cry1(TAG)::EGFP, 1mM	22837
	PER2::LUC + pSyn1.Cry1(TAG)::EGFP, 5mM	22838
	PER2::LUC + pSyn1.Cry1(TAG)::EGFP, 10mM	22839
	Cry1-Luc + pSyn1.Cry1(TAG)::EGFP, Vehicle	22840
	Cry1-Luc + pSyn1.Cry1(TAG)::EGFP, 1mM	22841
	Cry1-Luc + pSyn1.Cry1(TAG)::EGFP, 5mM	22842
	Cry1-Luc + pSyn1.Cry1(TAG)::EGFP, 10mM	22843
Figure 4	Cry1-Luc, jRCaMP1a + pSyn1.Cry1(TAG)::EGFP, 10mM, Bulk Data	22844
Figure 5	PER2::LUC + pSyn1.Cry1(TAG)::EGFP, Vehicle	22836
	PER2::LUC + pSyn1.Cry1(TAG)::EGFP, 10mM	22839
	Cry1-Luc + pSyn1.Cry1(TAG)::EGFP, Vehicle	22840
	Cry1-Luc + pSyn1.Cry1(TAG)::EGFP, 10mM	22843

SI References

1. van der Horst GT*, et al.* (1999) Mammalian Cry1 and Cry2 are essential for maintenance of circadian rhythms. *Nature* 398(6728):627-630.
2. Yoo SH*, et al.* (2004) PERIOD2::LUCIFERASE real-time reporting of circadian dynamics reveals persistent circadian oscillations in mouse peripheral tissues. *Proceedings of the National Academy of Sciences of the United States of America* 101(15):5339-5346.
3. Maywood ES*, et al.* (2013) Analysis of core circadian feedback loop in suprachiasmatic nucleus of mCry1-luc transgenic reporter mouse. *Proceedings of the National Academy of Sciences of the United States of America* 110(23):9547-9552.
4. Edwards MD, Brancaccio M, Chesham JE, Maywood ES, & Hastings MH (2016) Rhythmic expression of cryptochrome induces the circadian clock of arrhythmic suprachiasmatic nuclei through arginine vasopressin signaling. *Proceedings of the National Academy of Sciences of the United States of America* 113(10):2732-2737.
5. Ernst RJ*, et al.* (2016) Genetic code expansion in the mouse brain. *Nat Chem Biol* 12(10):776-778.
6. Smyllie NJ*, et al.* (2022) Cryptochrome proteins regulate the circadian intracellular behavior and localization of PER2 in mouse suprachiasmatic nucleus neurons. *Proceedings of the National Academy of Sciences of the United States of America* 119(4):e2113845119.
7. Maywood ES*, et al.* (2018) Translational switching of Cry1 protein expression confers reversible control of circadian behavior in arrhythmic Cry-deficient mice. *Proceedings of the National Academy of Sciences of the United States of America* 115(52):E12388-E12397.
8. Zhou H*, et al.* (2018) In vivo simultaneous transcriptional activation of multiple genes in the brain using CRISPR-dCas9-activator transgenic mice. *Nat Neurosci* 21(3):440-446.
9. Hastings MH, Reddy AB, McMahon DG, & Maywood ES (2005) Analysis of circadian mechanisms in the suprachiasmatic nucleus by transgenesis and biolistic transfection. *Methods Enzymol* 393:579-592.
10. Schindelin J*, et al.* (2012) Fiji: an open-source platform for biological-image analysis. *Nat Methods* 9(7):676-682.
11. Zielinski T, Moore AM, Troup E, Halliday KJ, & Millar AJ (2014) Strengths and limitations of period estimation methods for circadian data. *PLoS One* 9(5):e96462.
12. Brancaccio M, Maywood ES, Chesham JE, Loudon ASI, & Hastings MH (2013) A Gq-Ca2+ Axis Controls Circuit-Level Encoding of Circadian Time in the Suprachiasmatic Nucleus. *Neuron* 78(4):714-728.
13. Patton AP*, et al.* (2020) The VIP-VPAC2 neuropeptidergic axis is a cellular pacemaking hub of the suprachiasmatic nucleus circadian circuit. *Nat Commun* 11(1):3394.
14. Dorostkar MM, Dreosti E, Odermatt B, & Lagnado L (2010) Computational processing of optical measurements of neuronal and synaptic activity in networks. *J Neurosci Methods* 188(1):141-150.
